# A Programmable Delay Line-Based Measurement Methodology for CMOS LiDAR Receivers

**DOI:** 10.3390/mi17101149

**Published:** 2026-09-30

**Authors:** Bobin Seo, Sunkyung Lee, Somi Park, Sung-Min Park

**Affiliations:** Division of Electronic and Semiconductor Engineering, Ewha Womans University, Seoul 03760, Republic of Korea; qhqls28@ewha.ac.kr (B.S.); snklee20@ewha.ac.kr (S.L.); 1118som@ewha.ac.kr (S.P.)

**Keywords:** CMOS, LiDAR, programmable delay line, TIA, TVC

## Abstract

This paper suggests a novel measurement methodology to precisely evaluate the time-of-flight performance of LiDAR receivers (LRx) by using a CMOS programmable delay line-based time-to-voltage converter (PDL-TVC). Conventional LiDAR systems equip an external laser diode and a photodiode connected via bond wires; however, this causes not only severe signal distortions, but also unpredictable signal delays. In contrast, the proposed PDL-TVC employs a rising edge detector (RED) followed by an integrator and a peak-detect-and-hold (PDH) circuit to convert the time intervals into the output voltage pulses for precise distance estimation, thereby omitting the need of a costly LiDAR transmitter and the corresponding bond wires. In addition, a CMOS optoelectronic LiDAR receiver (COLR) is revealed to prove the proposed test methodology for LRx, comprising an on-chip avalanche photodiode to avoid bond wires, as well as an active feedback transimpedance amplifier followed by a Schmitt trigger to generate STOP signals to determine the time intervals from START pulses. Furthermore, a 4-to-10 decoder is added to provide externally selectable delay intervals of 1–10 ns, enabling the controlled test of the PDL-TVC operations. The proposed COLR chip is fabricated in a 180 nm CMOS process, occupying 312 × 659 μm^2^ for the core area. The PDL-TVC consumes 12 mW, with the programmable delay line consuming 374 µW under a 1.8 V supply. Measured results of the COLR confirms reliable on-chip signal generation and accurate characterization, thus demonstrating that the proposed measurement methodology using the PDL-TVC offers a precise solution for evaluating LiDAR receivers.

## 1. Introduction

**Figure 1:**
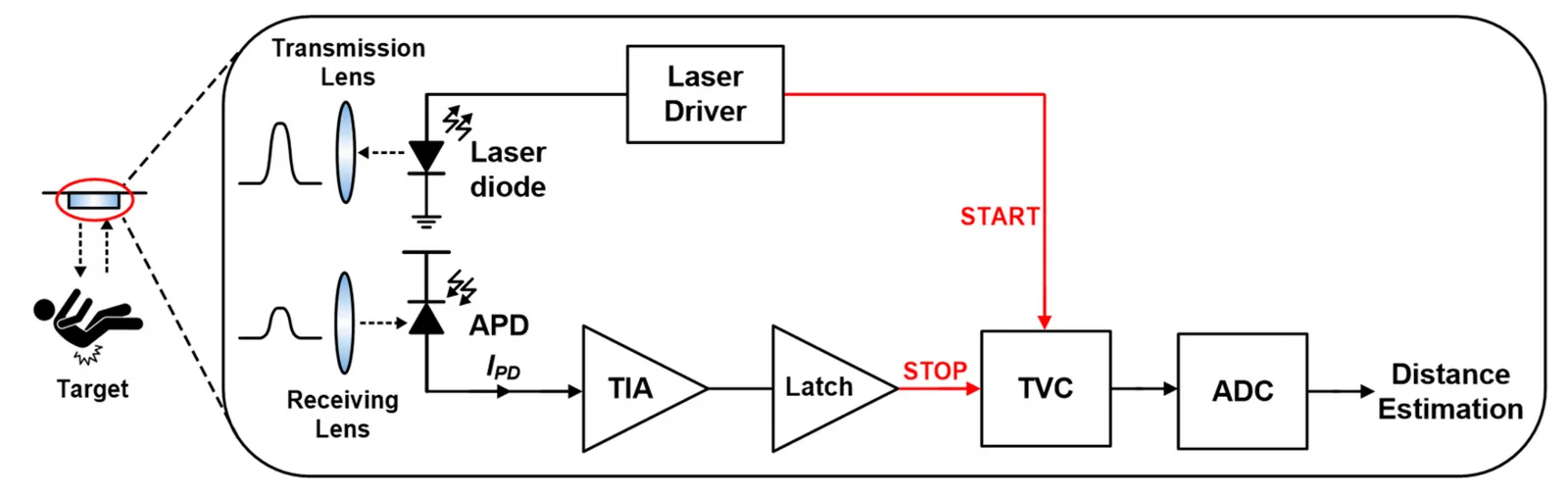
Block diagram of a conventional LiDAR sensor system.

Light detection and ranging (LiDAR) sensors have gained increasing significance in various applications such as autonomous vehicles, terrain exploration, and mapping [1,2,3,4,5,6]. Figure 1 shows the block diagram of a conventional LiDAR sensor system where the transmitter (Tx) is integrated with a LiDAR receiver (LRx) in the form of a system-on-chip (SoC). Yet, the optical devices such as laser diodes and photodiodes are separately mounted on the same PC-board (PCB) with the SoC. Then, the START signals from Tx must be transmitted through I/O pads, bond wires, and PCB traces. Therefore, these signal paths might cause severe signal distortion, deteriorating the evaluation accuracy of the LRx. Although several studies have demonstrated fully integrated LiDAR transceivers with on-chip photodiodes to alleviate this critical issue [7,8,9,10], laser diodes are still externally wire-bonded even with state-of-the-art CMOS technologies. In this paper, we present a novel measurement methodology that exploits a programmable delay line-based time-to-voltage converter (PDL-TVC). Since this configuration incorporates an on-chip programmable delay line (PDL) connected directly to the LRx, both the START and STOP signals can be generated and transmitted through the designated on-chip paths. Accordingly, the key contribution of this work is an on-chip electrical characterization methodology that provides programmable START-to-STOP intervals for evaluating LiDAR receiver timing performance while minimizing the feasible timing uncertainty introduced by external transmitters and off-chip interconnections.

This paper is organized as follows. Section 2 discusses the circuit operation, Section 3 presents the simulation results, Section 4 presents the measured results, and Section 5 concludes the study.

## 2. Circuit Description

**Figure 2:**
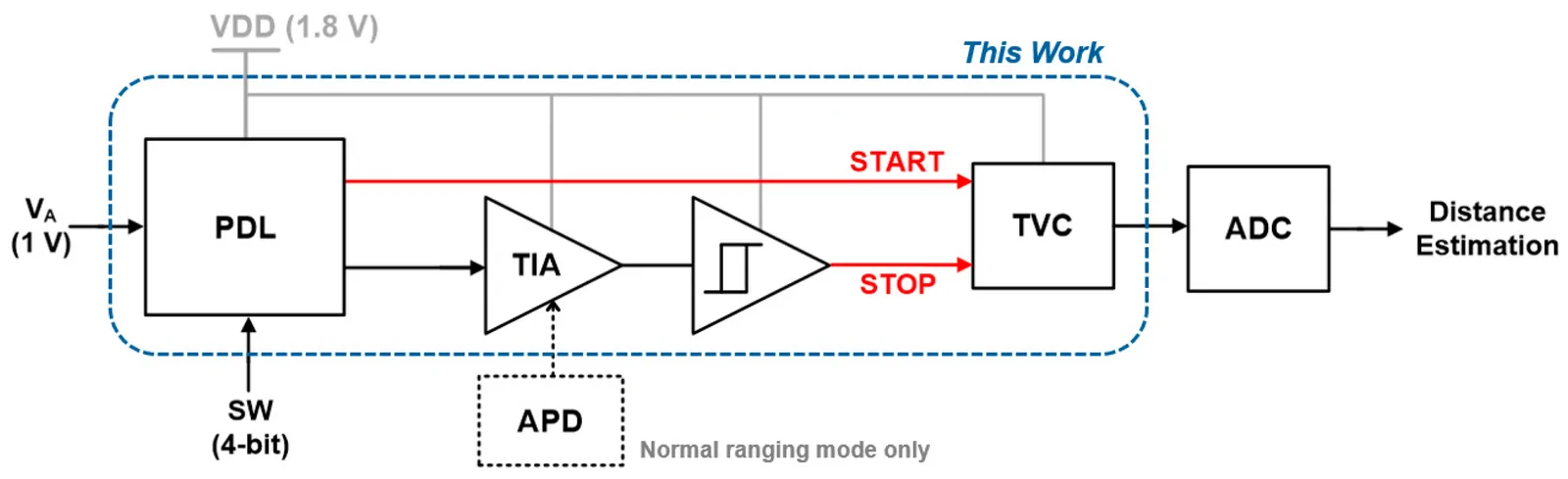
Block diagram of the proposed PDL-TVC.

Figure 2 depicts the block diagram of the proposed PDL-TVC circuit, where the PDL generates START and STOP pulses with controlled time intervals when a pulse signal (V_A_) is applied and the PDL is enabled by an external switch input (SW). Then, the START and STOP signals are fed into the TVC to generate voltage outputs that correspond to the time intervals between two pulses [11,12]. The subsequent signal-processing follows conventional post-processing steps, in which the analog outputs are digitized by a subsequent ADC. From the resulting binary code, the corresponding time interval can be accurately estimated for receiver characterization. Hereby, it should be emphasized that the proposed methodology is intended for the electrical characterization of the LiDAR receiver. During normal ranging operations in a typical LiDAR receiver, an optical pulse is emitted by an external LD and then the reflected signal is detected by an avalanche photodiode (APD) to generate STOP signals. In the proposed characterization mode, the external optical path is replaced with an internally generated timing stimulus with controlled START-to-STOP intervals, thereby allowing the timing response of a receiver to be evaluated without additional uncertainty from the external Tx and off-chip interconnections.

For the receiver path, an on-chip APD employs the same P^+^/N-well/deep-N-well (P^+^/NW/DNW) structure reported in [13,14]. Avalanche multiplication mainly occurs at the P^+^/NW junction, while the central P^+^ node is connected to the transimpedance amplifier (TIA) input to suppress slow substrate diffusion currents. The DNW helps improve near-infrared sensitivity by limiting carrier diffusion toward the P-substrate, and shallow trench isolation (STI) surrounding the junction is used as a guard structure to alleviate electric-field crowding and premature edge breakdown. Salicide blocking is applied to the light-sensitive P^+^ region to enhance optical absorption, whereas the P^+^ contact regions remain silicided to maintain a low series resistance [13,14].

**Figure 3:**
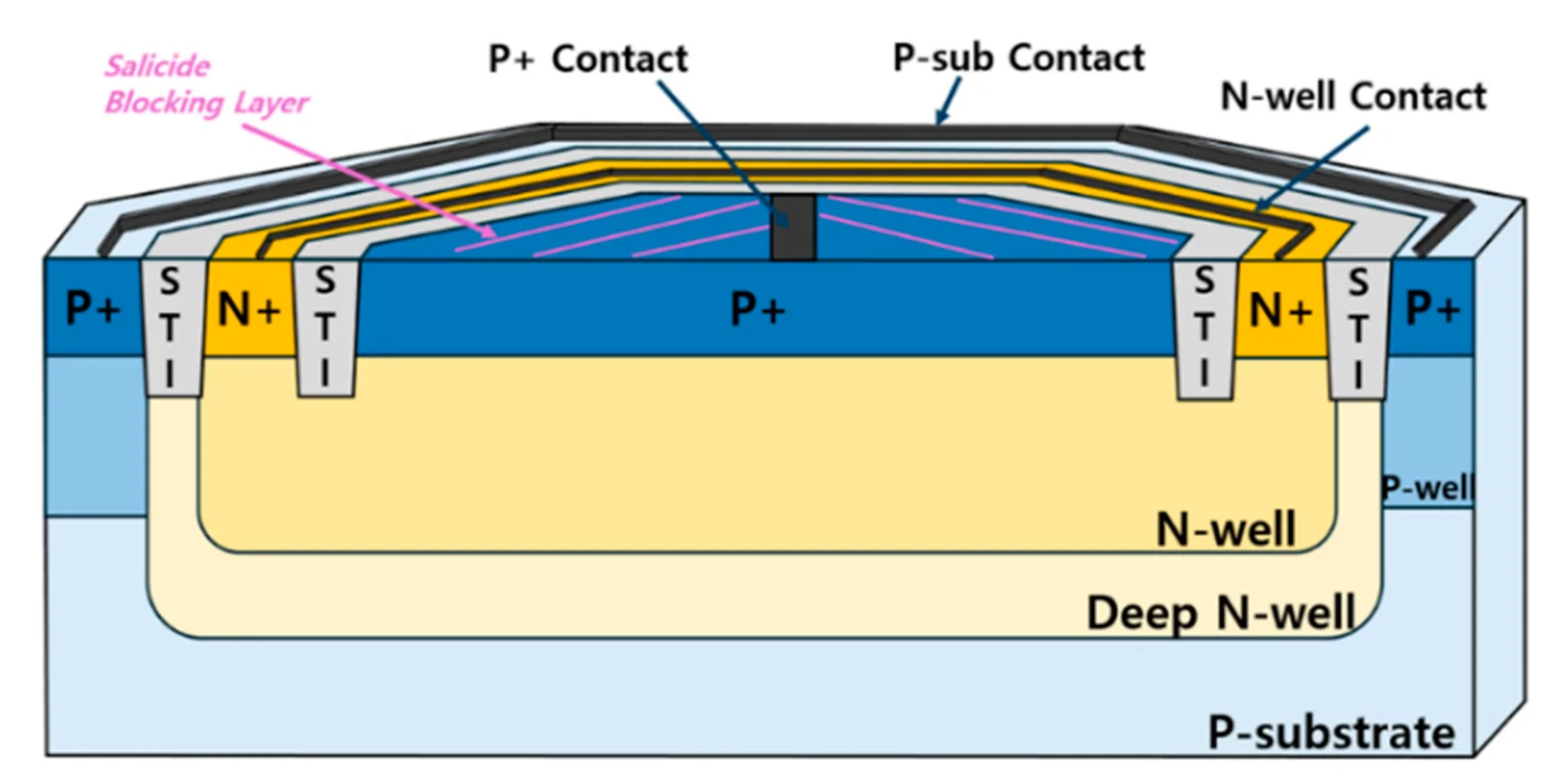
Cross-sectional structure of the on-chip P^+^/NW/DNW APD [13,14].

The APD is implemented with a 40 μm diameter octagonal active area to mitigate edge breakdown while maintaining high responsivity. The device achieves a responsivity of 4.16 A/W at a reverse bias of 10.5 V and a photodetection bandwidth of 1.7 GHz at 10.25 V [13,14]. The cross-sectional structure of the on-chip APD is illustrated in Figure 3.

**Figure 4:**
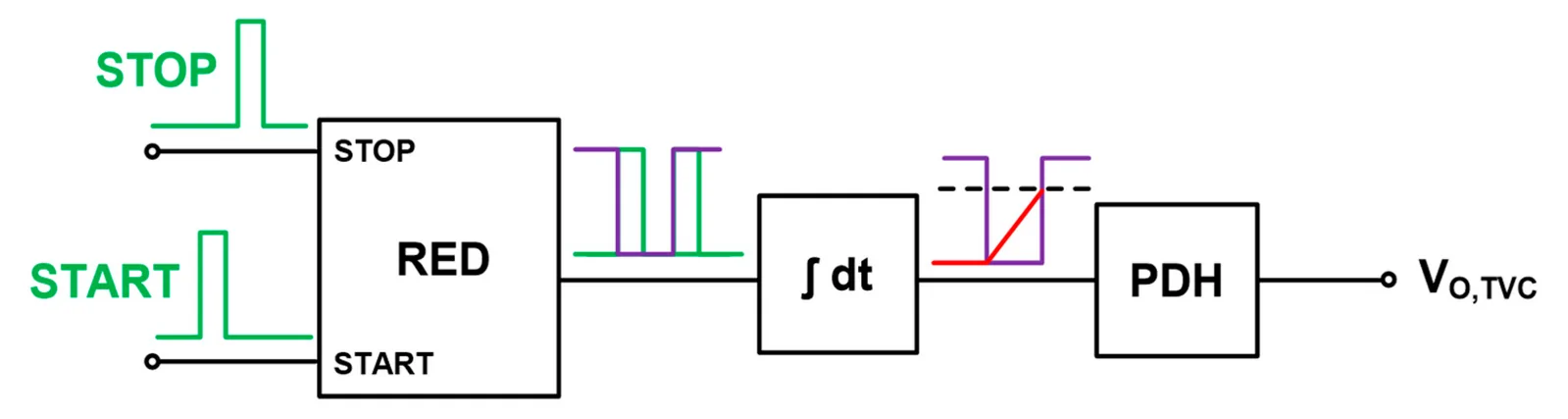
Block diagram of the proposed TVC.

Figure 4 illustrates the architecture of the proposed TVC circuit, which is a modified version of the previously reported time-to-voltage converter (T2V) [15]. Therefore, the START and STOP pulses generated by the PDL are first applied to a rising edge detector (RED). Using a combination of logic gates, the RED produces a signal that remains low only during the time interval between the rising edges of the START and STOP pulses. This control signal drives an integrator-based charger, which charges only during the low interval and yields a monotonically increasing voltage. Finally, a peak-detect-and-hold (PDH) circuit captures and stores the peak value of the charger voltage, which serves as the final analog output of the TVC [15,16].

### 2.1. Programmable Delay Line (PDL)

**Figure 5:**
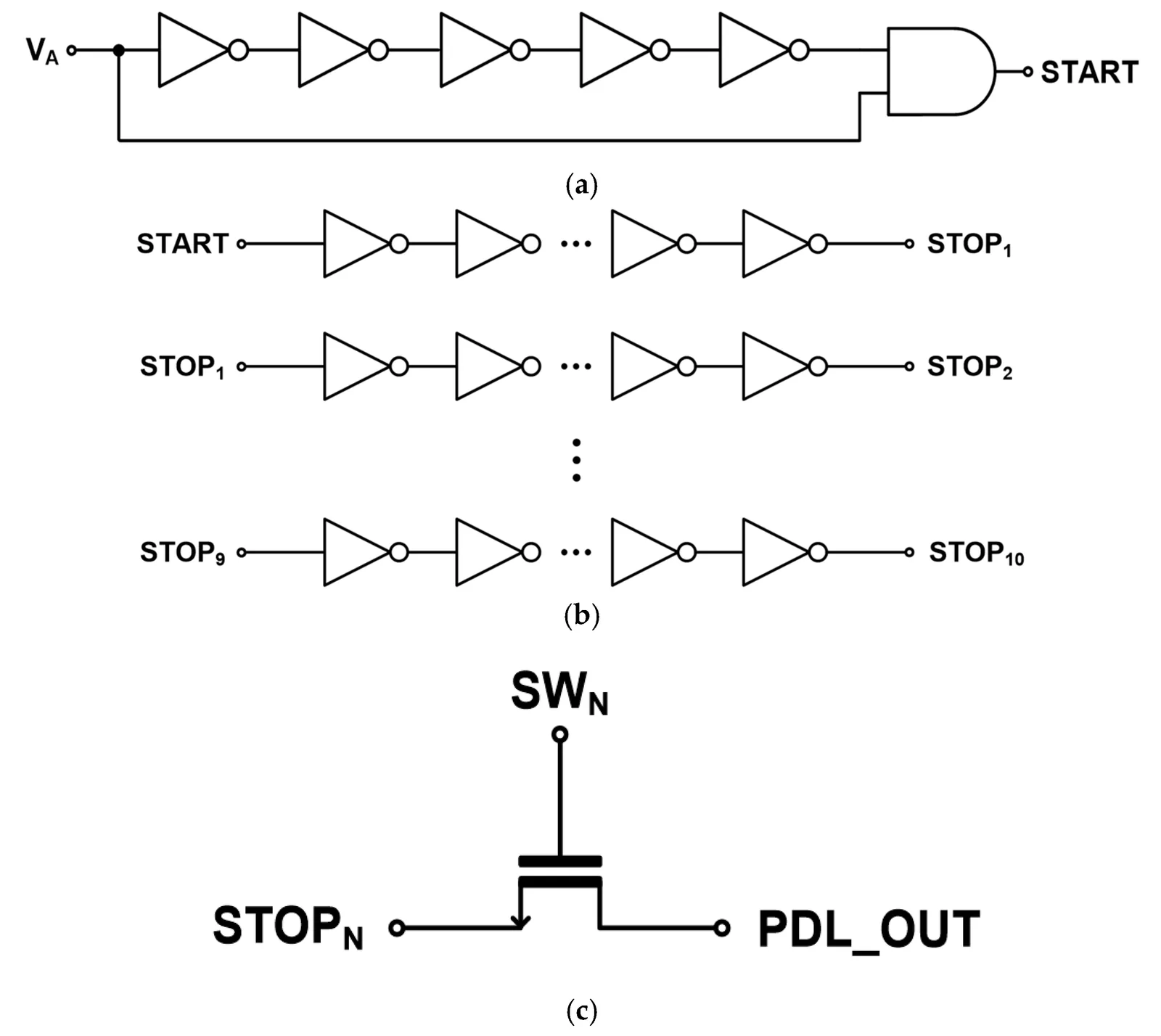
Block diagram of the programmable delay line (PDL): (**a**) START generator, (**b**) STOP generator, and (**c**) output switch.

Figure 5 shows the detailed operations of the PDL. First, the START generator is depicted in Figure 5a, where a 1 V rising-edge input pulse (VA) is combined through an AND gate with a delayed version of VA via a chain of inverters to generate the START signals.

Second, Figure 5b illustrates the STOP generator, in which the START pulses are delayed further through a series of inverter chains to produce ten distinct STOP signals. These chains are finely tuned so that every nine inverters introduce approximately 1 ns delay. It should be noted that a 4-to-10 decoder placed at the front end of the PDL selects a specific node in the delay chain according to the configuration of four external switch bits.

Depending on the decoded value, the circuit turns on an NMOS switch at the selected node, as depicted in Figure 5c and extracts the corresponding STOP pulse. This mechanism provides a reconfigurable timing resolution and supplies the STOP pulse to the TVC input, thus enabling precise and flexible time-to-voltage conversion.

### 2.2. Rising Edge Detector (RED)

**Figure 6:**
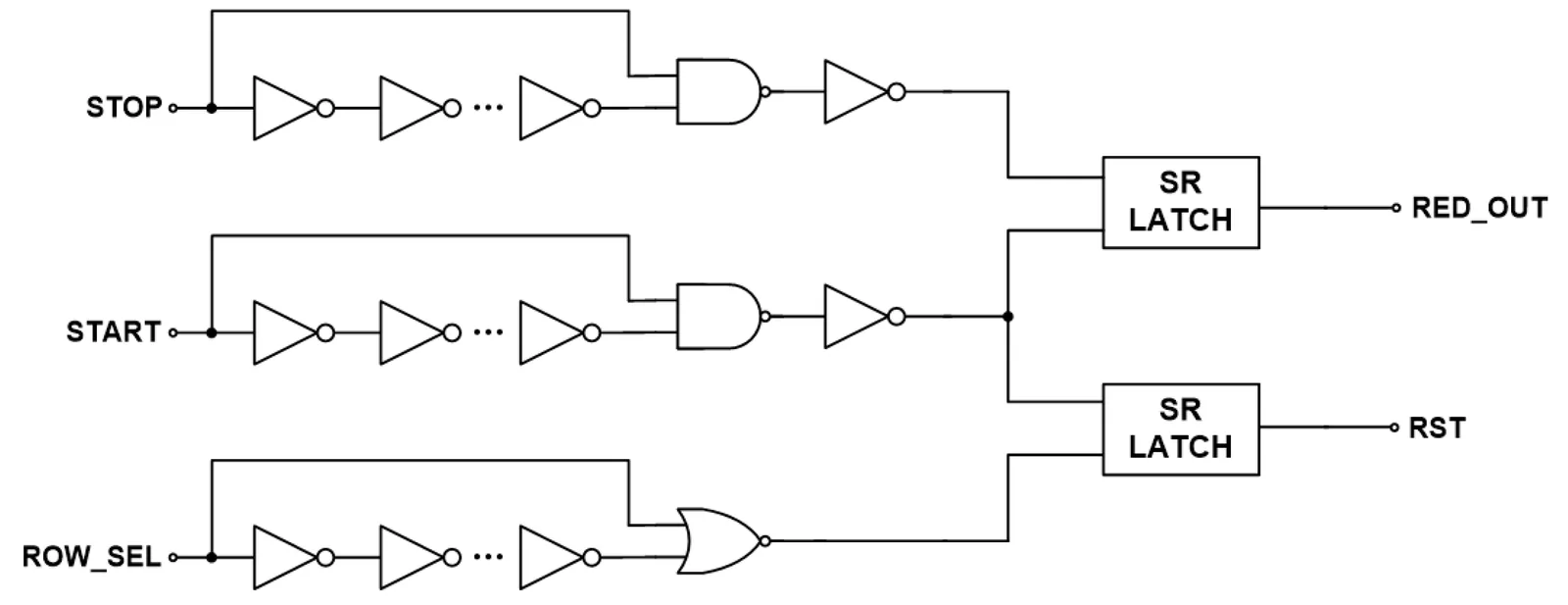
Block diagram of the RED.

Figure 6 shows the block diagram of the RED, where both the START and STOP signals, along with their slightly delayed pulses, are processed through AND operations to detect the rising edges. Then, these edges are applied to SR latches that ultimately produce the output control signals. These outputs remain low during the time intervals between the rising edges of the START and STOP pulses, while staying high otherwise.

In addition to the START/STOP-driven outputs, the RED also uses RST and ROW_SEL for array-level operations. ROW_SEL is a row-addressing signal that selects the row to be reset at the beginning of each measurement cycle, while RST resets the corresponding PDH output. The array-level addressing scheme itself is beyond the scope of the present single-channel characterization.

### 2.3. Integrator-Based Charger

**Figure 7:**
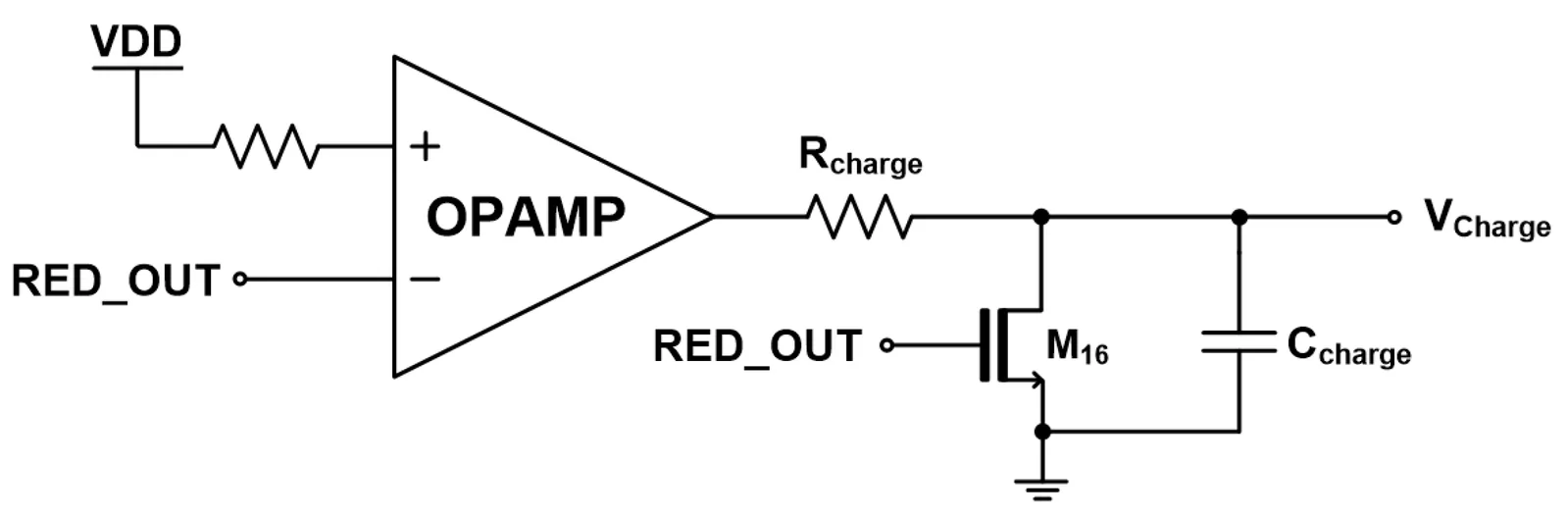
Schematic diagram of the integrator-based charger.

Figure 7 illustrates the schematic diagram of the integrator-based charger that employs an operational amplifier, an NMOS transistor, and a capacitor (*C*_charge_) to integrate the input signal. During an active charging interval, the internal voltage (*V*_Charge_) increases approximately linearly with time, while it discharges after the charging interval is terminated. The front-end op-amp, as an input stage, operates as a switch where the positive input is connected to VDD to function as a passive switch. The following resistor (R_charge_) isolates the op-amp output from the load capacitor, thereby improving the linearity of the charging circuit. The NMOS transistor (M_16_) helps to remove the DC offset voltage, thus allowing the entire circuit to interact effectively and produce more accurate outputs.

### 2.4. Peak Detect and Hold (PDH)

**Figure 8:**
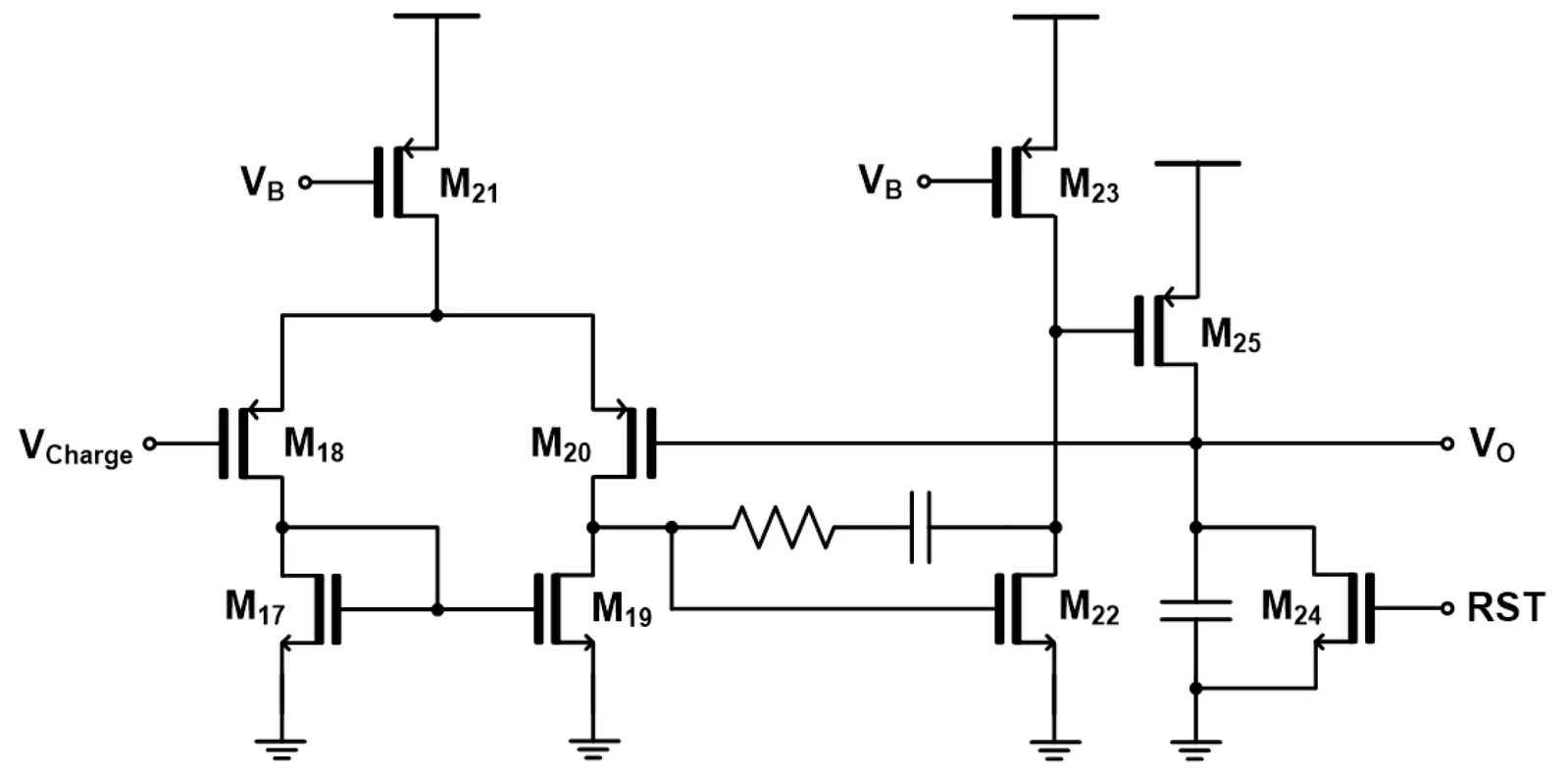
Schematic diagram of the PDH.

Figure 8 depicts the schematic diagram of the PDH circuit that receives the charging voltage (*V*_Charge_) and generates the final TVC output (*V_O_*). The voltage corresponding to the charging interval is detected and held, while the PDH circuit produces a level-shifted output voltage. Since the PDH output is directly proportional to the charged level, the analog voltage inherently encodes the time interval between the START and STOP pulses, which corresponds to the measured time-of-flight (or target distance) information.

## 3. Layout and Simulation Results

Post-layout simulations were conducted by using the device models of a standard 180 nm CMOS process. The DC analysis reveals that the proposed COLR operates with a 1.8 V supply voltage, consuming 12 mW (with the PDL dissipating 374 µW only).

**Figure 9:**
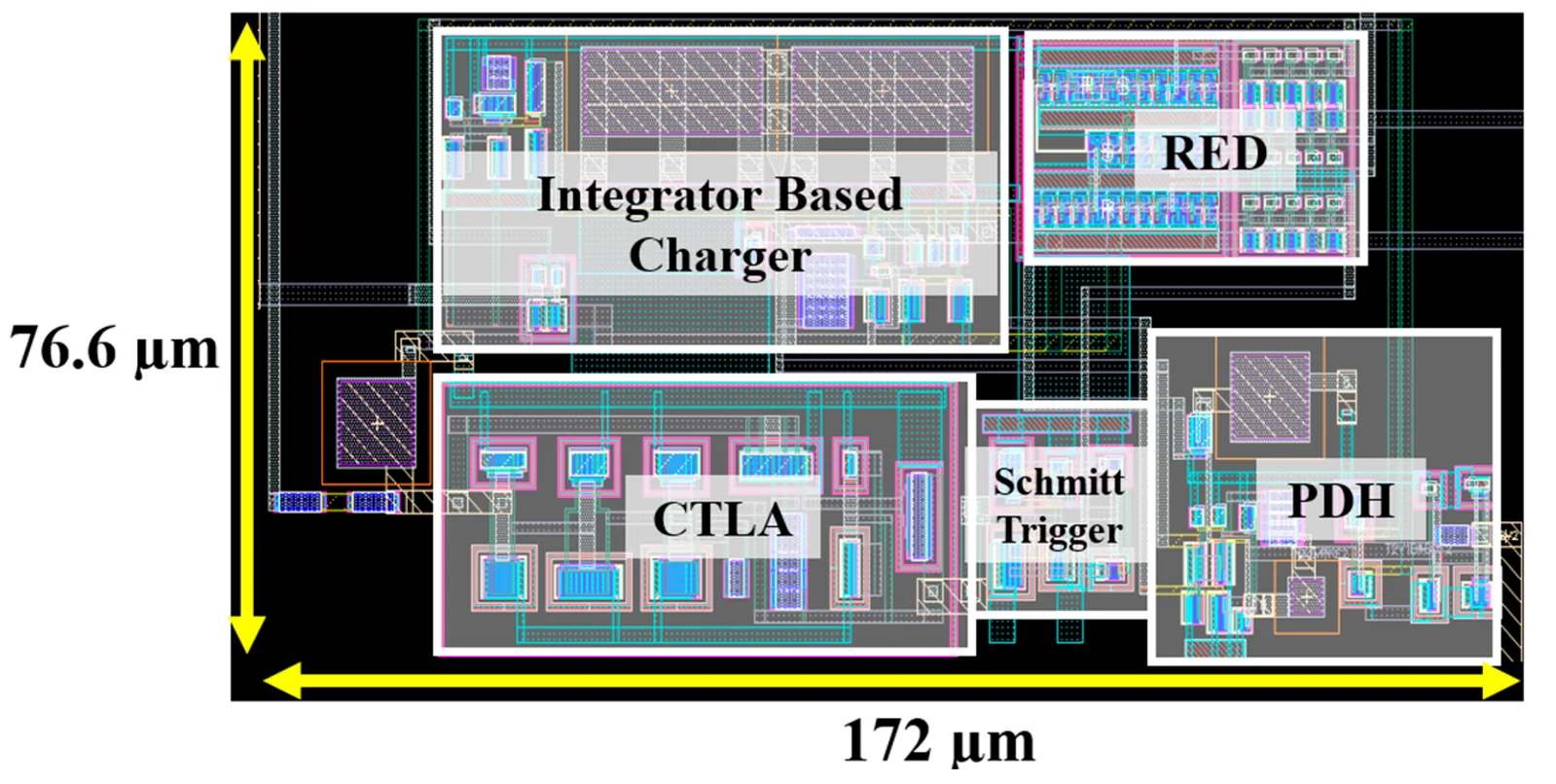
Layout of the subsequent circuit blocks following the PDL in the PDL-TVC.

Figure 9 shows the layout of the circuit blocks following the PDL in the PDL-TVC. These blocks consist of a CMOS transimpedance-limiting amplifier (CTLA) [17], a Schmitt trigger, and a TVC comprising the RED, charger, and PDH. The overall layout occupies an area of 76.6 × 172 µm^2^.

**Figure 10:**
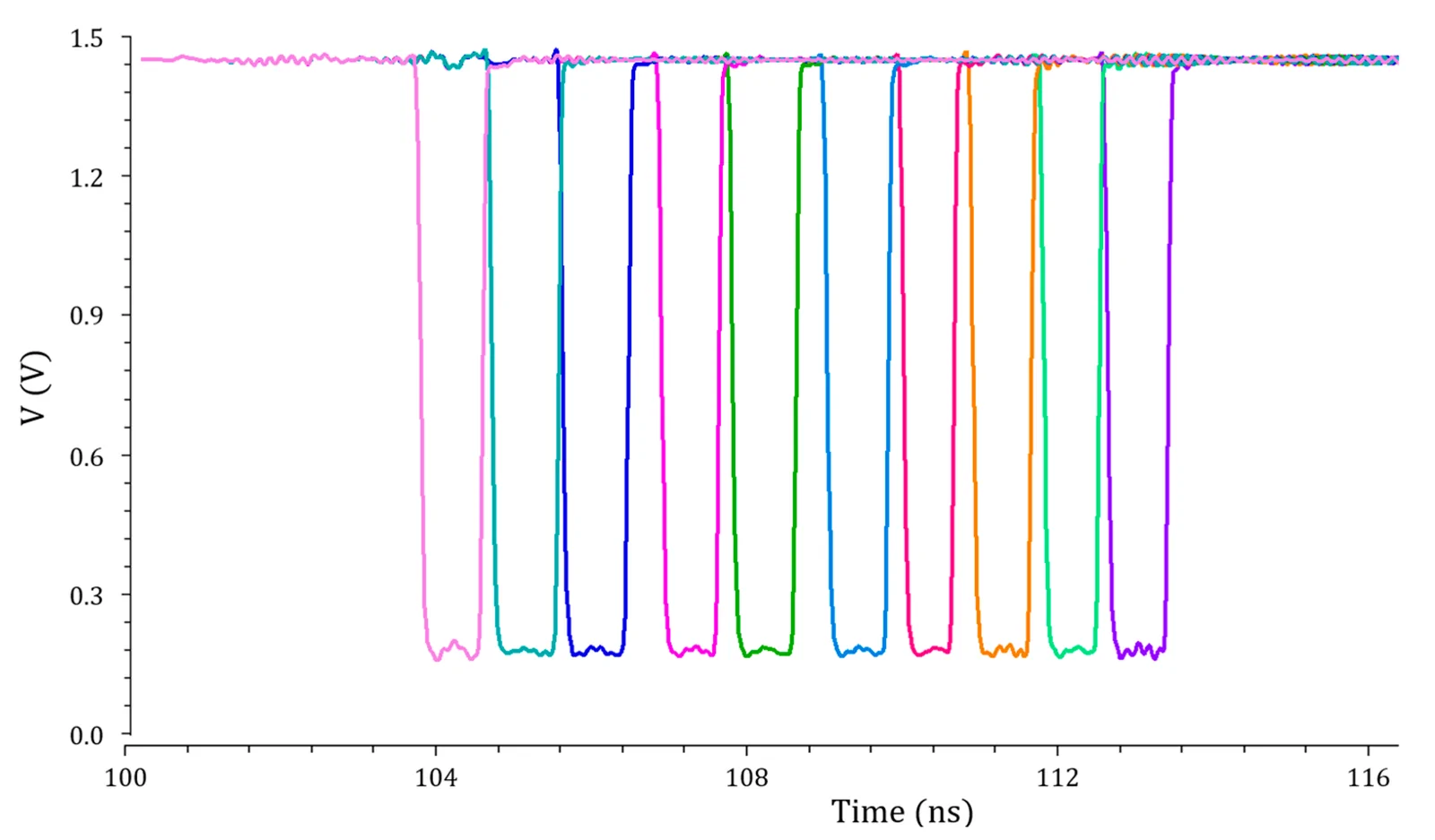
Simulated output waveform of pulses generated by the PDL, with STOP selection ranging from 1 to 10 ns, respectively.

Figure 10 presents the simulated STOP signals generated by the PDL. With the input signal applied after a 100 ns delay, ten STOP pulses are generated with a 1 ns interval.

While the pulses exhibit minor ripples, the key operations of the circuit are edge detection for the time-to-voltage conversion. Furthermore, the rising and falling edges of the pulses are preserved with no evident spreading.

**Figure 11:**
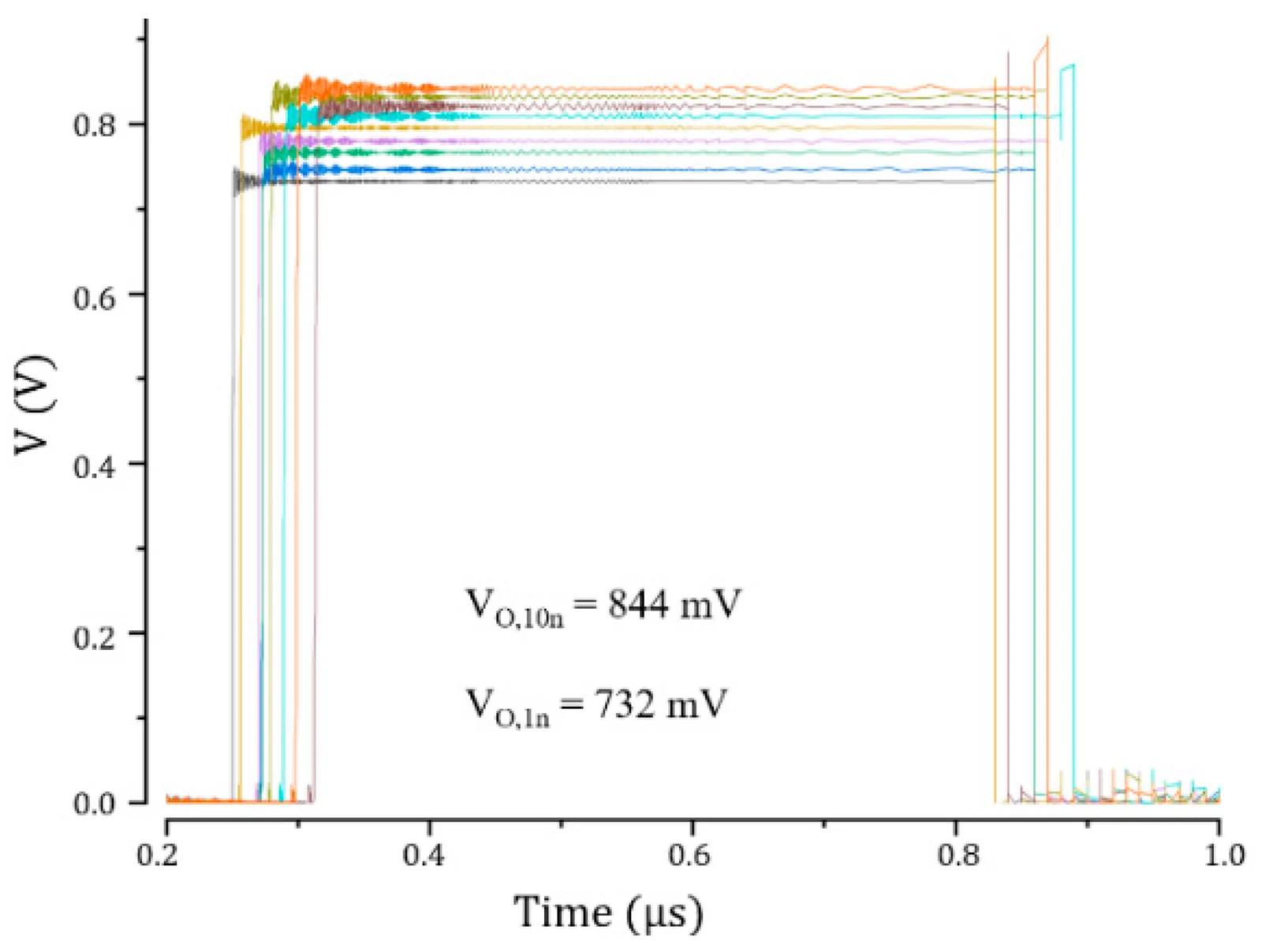
Simulated output waveform of the proposed PDL-TVC corresponding to time intervals of 1–10 ns, respectively.

Figure 11 shows the simulated output waveforms of the proposed COLR for the time intervals ranging from 1 to 10 ns. The simulated output voltages are 732 mV_pp_ at 1 ns, 780 mV_pp_ at 5 ns, and 844 mV_pp_ at 10 ns, respectively. These results verify the linear characteristic of the time-to-voltage conversion with the PDL-TVC.

For comparison, a conventional off-chip LiDAR signal path was modeled by using lumped equivalent circuits representing a LD, an APD, and their associated interconnection parasitic components. In its Tx path, the LD was represented by an equivalent resistance of 60 Ω and a capacitance of 850 fF, while the off-chip interconnection was modeled by utilizing a 2 nH series inductance and a 400 fF pad capacitance. In the Rx path, the off-chip APD was modeled by using a photocurrent source with a junction capacitance of 1.5 pF. Furthermore, a 25 Ω series resistance, a 2 nH interconnection inductance, and a 400 fF pad capacitance were included to represent the parasitic effects between the APD and the receiver chip. The APD capacitance was additionally varied from 1 to 3 pF to investigate its influence on the receiver timing response.

**Figure 12:**
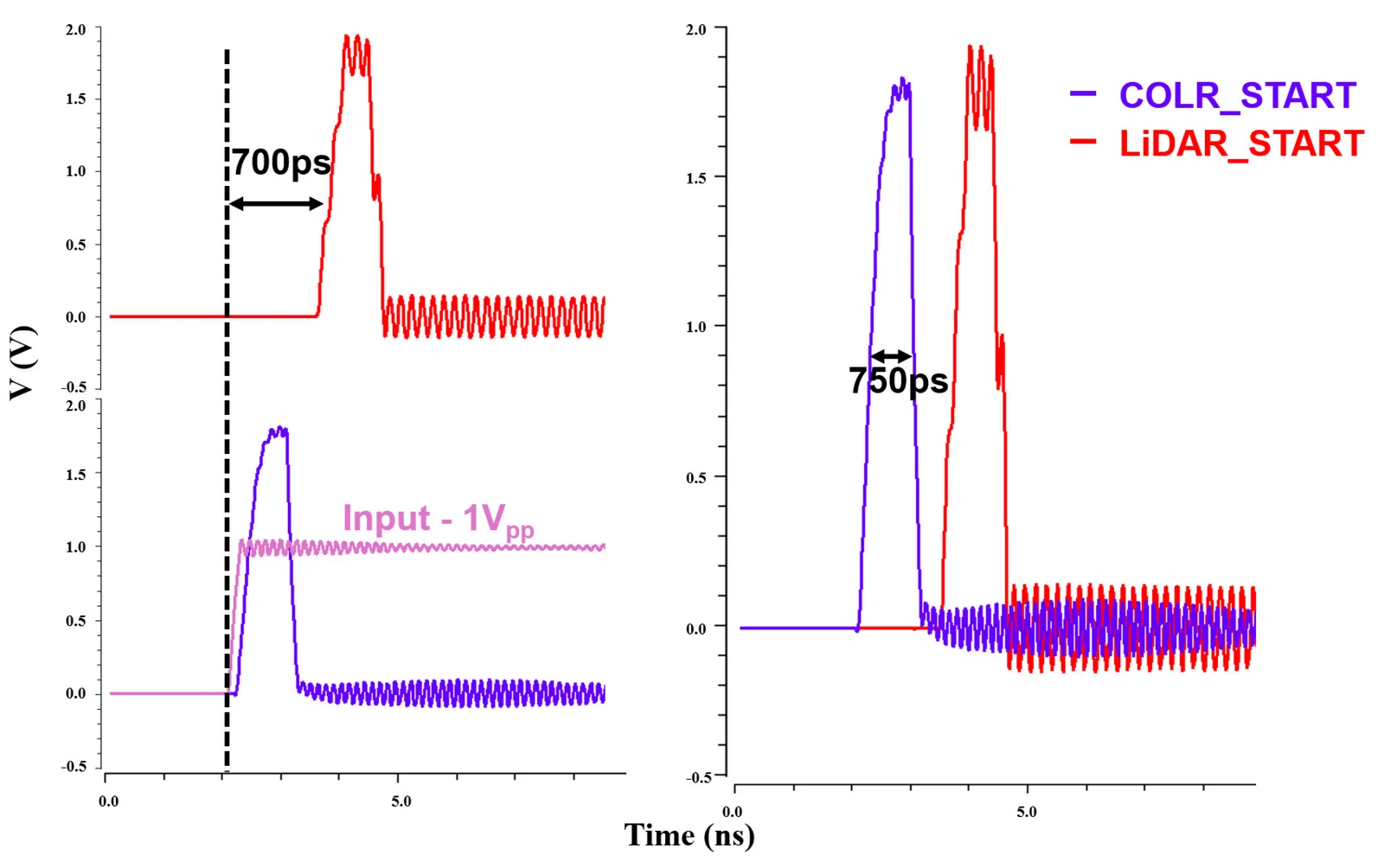
Comparison of simulated START signals generated by the proposed COLR with a conventional LiDAR system.

Figure 12 reveals the comparison of the START generation timing between the proposed COLR and the conventional off-chip LiDAR equivalent model. It is clearly seen that the COLR generates the START signal with negligible delay with a 1 V input amplitude, while the conventional LiDAR system (with an equivalent modeling in [18]) exhibits a significantly delayed START signal that might be attributed to uncontrollable parasitic effects such as bond wires.

The simulation results in Figure 12 confirm that these undesirable parasitic components introduce additional timing uncertainty, i.e., 700 ps delay, which would degrade the evaluation accuracy of the proposed receiver architecture. In contrast, the internally generated START signals from the on-chip PDL can neglect the obscure timing uncertainty, thus allowing a precise validation of any LiDAR receiver.

**Figure 13:**
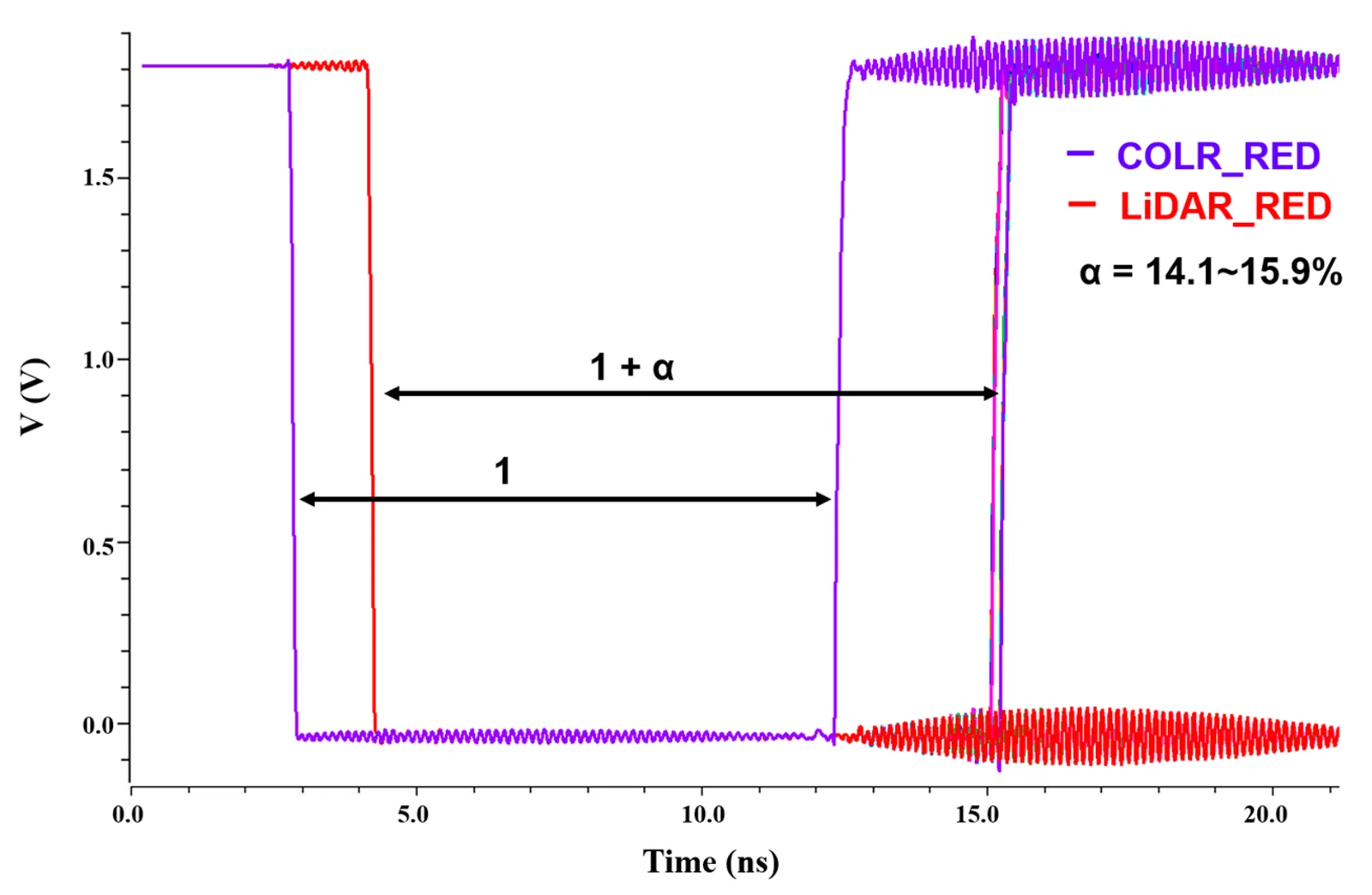
Comparison of simulated RED output signals generated by the proposed COLR with a conventional LiDAR system.

Figure 13 compares the simulated RED output signals of the COLR with that of a conventional LiDAR system, where the RED output is responsible for the charging operations. The simulations were configured with a 10 ns time interval for both the COLR chip and the LiDAR model, in which the purple waveform represents the RED output of the COLR chip. It is clearly seen that the RED output of the LiDAR model is generated later owing to the delayed START signal. Furthermore, timing variations are observed in the RED output signal at the STOP transition for the LiDAR model. These variations are certainly attributed to several undesirable effects such as off-chip APD variations and bond wires, thus leading to changes in the slope parameter, i.e., α. With a small variation (from 1 pF to 3 pF) of the off-chip APD parasitic capacitance, the value of α varies by approximately 14.1~15.9%. Therefore, the final RED output signals show significant delay correspondingly. Even when the APD capacitance exceeds 3 pF, the RED output signal fails to operate properly.

## 4. Chip Fabrication and Measurements

**Figure 14:**
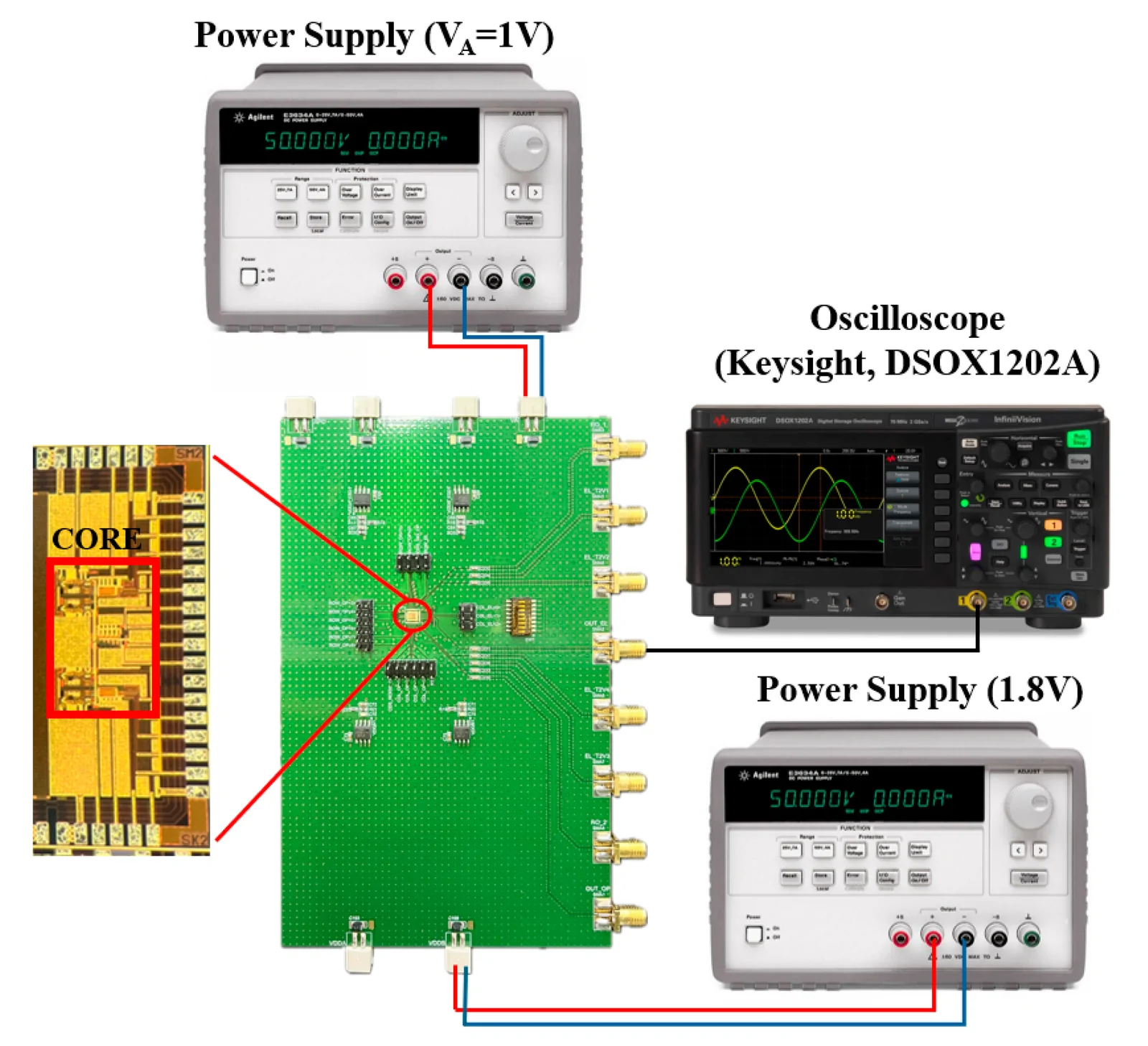
Chip photo of the COLR and its test setup.

Test chips of the proposed architecture were fabricated in a standard 180 nm CMOS process. Figure 14 shows the chip micrograph and the measurement setup of the proposed COLR. The entire chip, including the I/O pads, occupies the area of 2.5 × 2.0 mm^2^, while core circuit occupies 312 × 659 μm^2^. A 1.8 V supply voltage and a 1 V input (V_A_) of the PDL are provided by two separate external DC power supplies. The output responses are measured by using an oscilloscope (Keysight DSOX1202A).

**Figure 15:**
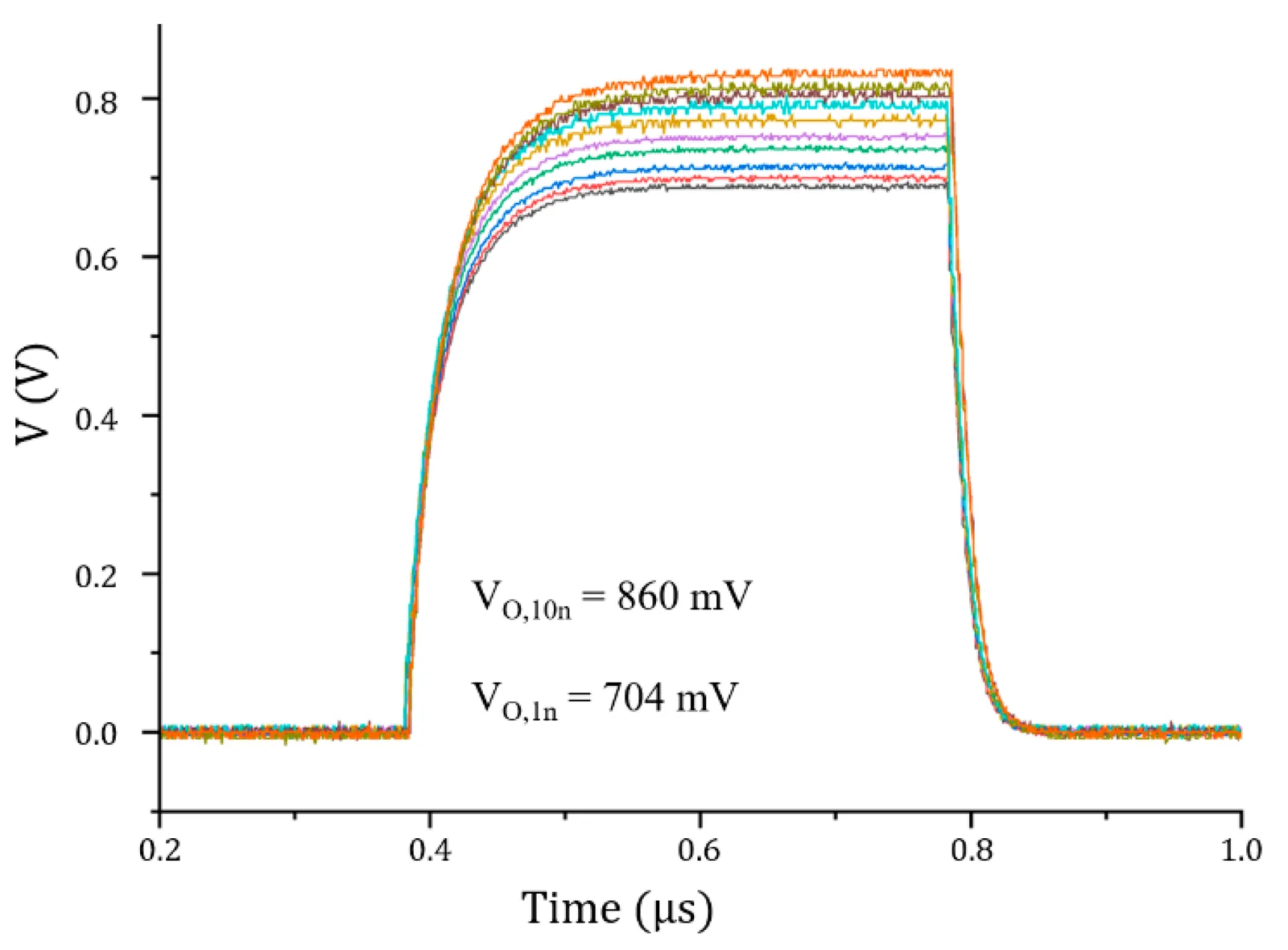
Measured output waveform of the proposed PDL-TVC corresponding to time intervals of 1–10 ns, respectively.

**Figure 16:**
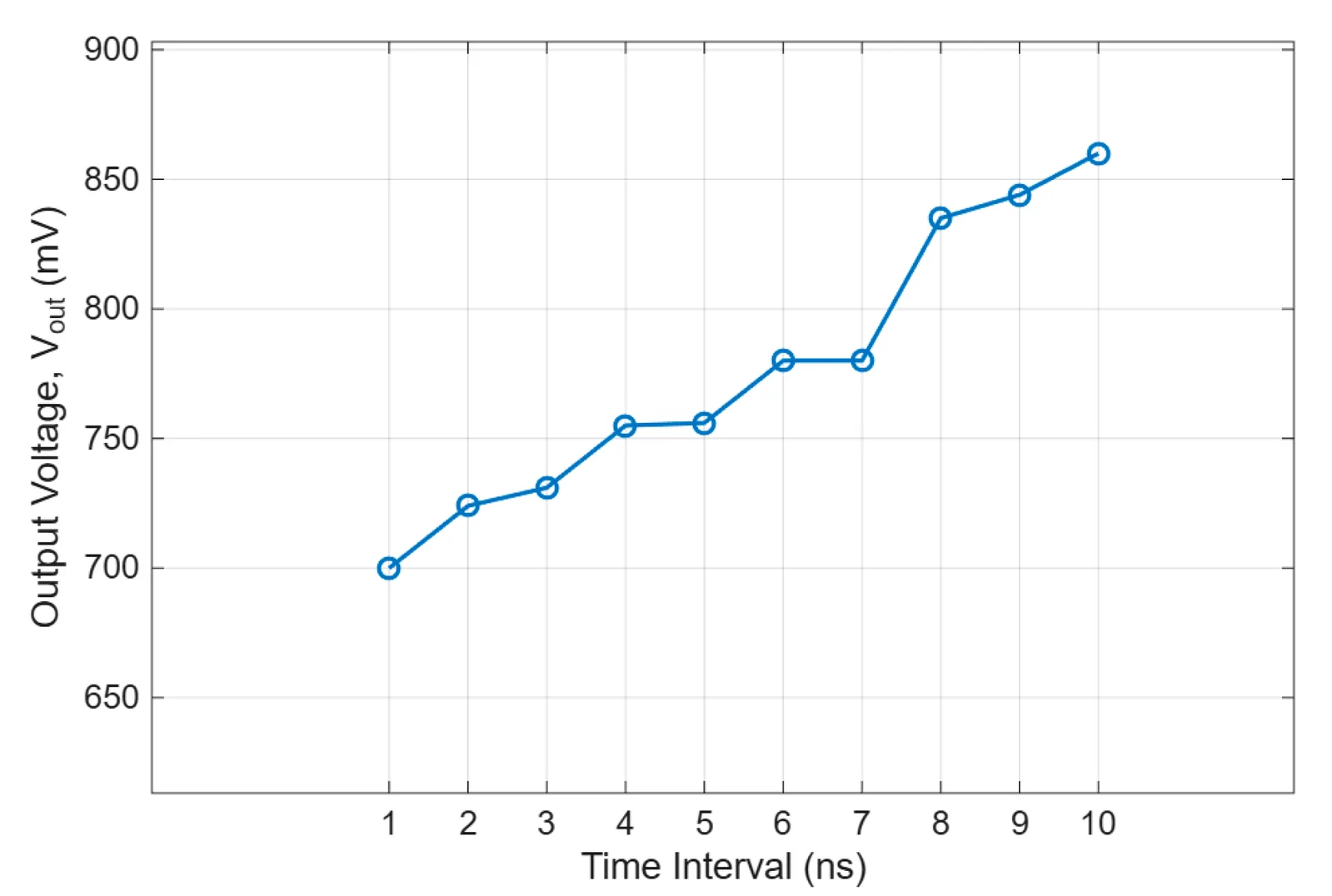
Measured output voltage of the proposed PDL-TVC as a function of the programmed time intervals from 1 to 10 ns.

Figure 15 demonstrates the measured final output of the proposed core circuit for time intervals ranging from 1 to 10 ns. The measured voltages are 704 mV_pp_ at 1 ns, 756 mV_pp_ at 5 ns, and 860 mV_pp_ at 10 ns, demonstrating a wider output range than the simulation results. This deviation is likely attributable to parasitic effects introduced by the bonding wires during measurement. Nevertheless, the measured results consistently confirm the proportional relationship between the output voltage and the applied time interval. This relationship is further illustrated in Figure 16, which depicts the measured output voltages as a function of the programmed time interval from 1 to 10 ns. The output voltage generally increases with the programmed time interval, confirming the time-to-voltage conversion characteristics of the proposed PDL-TVC.

Table 1 provides a performance comparison of various ToF systems from a measurement perspective.

Ref. [19] presents a chipset consisting of a receiver (Rx) and a TDC. This system improves accuracy through time-walk compensation. However, since the TDC, Rx, and APD are implemented as separate blocks, additional integration is required, which may introduce extra noise through bonding wires and interconnections. Furthermore, the measurement setup is not straightforward as external equipment is necessary.

Ref. [20] describes a discrete Rx and a discrete TDC implemented on a PCB. While this system demonstrates the advantage of long measurement range, calibration is performed in the FPGA using algorithms, which complicates the setup.

Ref. [21] demonstrates a system composed of a TDC and a PLL. Although this architecture is robust against PVT variations, it suffers from relatively large power consumption and chip area. Furthermore, no detailed measurement results are provided.

Ref. [22] employs a TDC array combined with an on-chip ring oscillator. The mutually coupled ring oscillators eliminate the need for explicit calibration. However, the overall chip size is relatively large even in a 65 nm process.

Ref. [23] introduces a TDC-based system. Although the START and STOP signals are externally generated, the setup is relatively simple. Nevertheless, this still requires significant power and area. In comparison, the proposed PDL-TVC system generates both START and STOP signals internally without relying on external instruments, thereby achieving a highly simplified measurement setup. Furthermore, it features significantly reduced chip and low power consumption.

## 5. Conclusions

This paper presented a PDL-based time-to-voltage converter (PDL-TVC) for short-range LiDAR receiver applications. To ensure a reliable evaluation of receiver performance, an on-chip PDL was incorporated to generate both START and STOP signals and deliver them through on-chip paths. This approach eliminates distortions and losses caused by I/O pads and bond wires, while simplifying the measurement setup. To enable reliable testing, two TVC channels are driven by a shared PDL with externally selectable delay intervals provided by a 4-to-10 decoder. The test chip was fabricated in a standard 180 nm CMOS process and measured under a 1.8 V supply. The proposed TVC achieves low power consumption and compact chip area. These attributes render the TVC an effective and energy-efficient solution for integrated LiDAR receiver ICs targeting short-range indoor sensing.

## Figures and Tables

**Table 1:** Performance comparison of recently reported receiver and timing-conversion circuits for ToF LiDAR systems.

Parameters	[19]	[20]	[21]	[22]	[23]	This Work
Application System	Pulsed dToF	Pulsed dToF	LiDAR Sensor	dToF	LiDAR Sensor	LiDAR Sensor
Architecture	Rx + TDC chipset	Integrated Rx + TDC on PCB	TDC + PLL	TDC array + RO	TDC	TVC channel+ PDL
Technology	CMOS 350 nm	Discrete device	CMOS 180 nm	CMOS 65 nm	CMOS 180 nm	CMOS 180 nm
Supply Voltage (V)	3.3	-	1.8	1.2	1.8	1.8
Power Dissipation (mW)	180 (Rx) 150 (TDC)	30	13.6 (TDC) 2.90 (PLL)	0.5	20.9 (per channel)	12.0 (per channel) 0.374 (PDL)
Chip Area (mm^2^)	4 (Rx) 10 (TDC)	0.04 (active area)	0.126	0.55 (RO) 1.69 (TDC)	0.225	0.206
Stimulus	External	Internal	N/A	Internal	External	Internal
Calibration Strategy	Time-walk compensation	In situ @ FPGA	Dynamic element matching	Mutually coupled RO	-	-
External Equipment Dependence	Yes	Yes	N/A	No	Yes	No
Setup Simplification	No	No	N/A	Yes	Yes	Yes

## Data Availability

The original contributions presented in this study are included in the article. Further inquiries can be directed to the corresponding author.

## Abbreviations

The following abbreviations are used in this manuscript:

COLR	CMOS Optoelectronic LiDAR Receiver
CTLA	CMOS Transimpedance-Limiting Amplifier
LRx	LiDAR Receiver
PDH	Peak Detect and Hold
PDL	Programmable Delay-Line
PDL-TVC	Programmable Delay Line-Based Time-to-Voltage Converter
RED	Rising Edge Detector
TIA	Transimpedance Amplifier
TVC	Time-to-Voltage Converter
